# Decentralisation; The Question of Management Capacity: A Response to Recent Commentaries

**DOI:** 10.15171/ijhpm.2016.134

**Published:** 2016-10-04

**Authors:** Jalal Mohammed, Nicola North, Toni Ashton

**Affiliations:** Health Systems Section, School of Population Health, The University of Auckland, Auckland, New Zealand.

Whilst decentralisation as an instrument of healthcare reform remains popular, commentaries^1-5^ to our paper titled “Decentralisation of health services in Fiji: A decision space analysis”^6^ highlight the complexity in understanding decentralisation, with the significant body of research on decentralisation lacking consensus on its definition,^7^ differing on what constitutes decentralisation,^7-11^emphasising different theoretical underpinnings and frameworks,^7,12-14^ and reporting varying applications and outcomes of decentralisation.^15^ This is a consequence of widely varied health systems in which decentralisation has been initiated. Viewing decentralisation on a continuum, with centralisation and decentralisation on polar ends, demarcated through broad linear categories (deconcentration, delegation, devolution and privatisation)^12,13^ has been useful in understanding decentralisation. However, these categories are not analytical in nature, posing problems in capturing degrees of decentralisation and for making useful comparisons. Several analytical frameworks, including the ‘decision space approach’^16^ were posited to empirically analyse decentralisation and address gaps in the decentralisation literature.

Our article, limited in scope, employed the widely applied decision space framework in understanding Fiji’s decentralisation of a particular function, service delivery. Application of the decision space framework was suited to Fiji’s hierarchal health system, which is inherently vertical in nature. Decentralisation initiatives may not fit neatly into analytical frameworks as illustrated in our analysis, and the use of alternative frameworks such as the arrows framework^7^ to examine Fiji’s decentralisation initiative would increase understanding. Indeed, in a preceding study,^17^ we examined both decentralisation initiatives in Fiji from a functional perspective. That analysis has led to a forthcoming publication,^18^ where we explore some of the reasons why the current decentralisation initiative has been considered successful by the Fijian government.

In the case of Fiji, the least imposing form of decentralisation (deconcentration) was applied, entailing shifting workload from the tertiary hospital to the peripheral health centres, without a commensurate transfer of authority. There are arguments that deconcentration without a transfer of authority should not be considered decentralisation as the organisation continues to behave like a centralised system.^10,19^ The decision space analysis of Fiji’s decentralisation initiative supports this view, revealing a system that remains centrally controlled. The Fijian health system reflects a U Form organisation^2^ whereby health services are delivered through a network of government owned facilities, controlled by divisional managers who liaise with subdivisions to ensure that a minimum standardised package of health services is delivered. While this has the benefit of realising economies of scale, there is growing evidence that countries could better benefit from a mix of centralisation and decentralisation strategies, taking advantage of efficiencies gained from centralisation of certain functions and decentralisation of others.^8,14,20-22^ In Fiji, such strategies would allow for both the efficiency of a centralised system while allowing greater flexibility in response to local communities. However, this would require substantial decentralisation (beyond deconcentration) taking hold.

In his commentary,^5^ Peckham noted that an examination of both vertical and horizontal relationships would better explain decision space at the decentralised health centres. We agree that horizontal relationships affect the degree of decision space and warrants further exploration in Fiji’s case. In our article, we privileged the vertical relationship, as in Fiji’s health system relationships are mainly vertical in nature. For example, service delivery is designed to be hierarchal, with nursing stations at the lowest level and hospitals at the highest level. In Fiji, there are potential benefits to be gained from horizontal decentralisation in the area of intra-divisional relationships. Our study reveals some change in horizontal relationships as a result of decentralisation. The six decentralised health centres, categorised from level C (lowest) to level A (highest), vary in the diagnostic services that they offer, resulting in sideways (horizontal) referrals from smaller to larger health centres for diagnostic services. For referrals to divisional hospitals, there is a dependent relationship between the health centre and the divisional hospital, whereby health centres have to ensure that beds are available in divisional hospitals before referring patients. If beds are not available, the patient is held at the health centre until a bed becomes available. However, in most respects the decentralised health centres offer limited autonomy in practitioner practice, and remain closely overseen through quarterly rotation of medical officers between the decentralised health centres. Additionally, the increased utilisation of the six health centres has meant that practitioners spend under five minutes per patient which has left little room for deviation from standard operating guidelines.^6^

Management capacity is considered vital to the success of decentralisation and strengthening is required at lower levels in order for decentralisation to succeed.^12,23-25^ However, as evident in Fiji’s experience, the creation of decision space may not result in increased management capacity. Fiji’s first decentralisation initiative was hampered by several issues, including having only three health service managers with the qualifications and experience to take on decision-making responsibilities.^26^ This meant decision space created at decentralised levels could not be realised, impacting not only on implementation and outcomes, but on the success of the decentralisation initiative. Therefore, whilst it is important to have an understanding of the degree of decentralisation, a measure of management capacity pre and post decentralisation would strengthen decentralisation frameworks. From a developing country perspective where resources and skills are limited, and decentralisation is used as a means to improve not only the responsiveness of health service delivery but to improve efficiencies, understanding management capacity is integral to the decentralisation process. Without management capacity, the creation of decision space may not enable the benefits of decentralisation to be realised, illustrated in Fiji’s case.

The debate around the effectiveness of decentralisation will continue and is unlikely to provide concrete answers for countries seeking to emulate models of successful implementation. There is no one-size-fits-all solution when it comes to decentralisation^25^ and indiscriminate adoption has led to negative outcomes, unintended consequences and a questioning of the benefits of decentralisation. Fiji’s experience reveals that countries need to follow their own path in decentralising, adapting to local environment and local needs in order to increase its chances of success.^18^

In spite of growing interest in analysing decentralisation, there has been less focus on understanding how decentralisation impacts major health system goals of equity, efficiency, and access. Our decision space analysis forms part of a larger study examining the impacts of decentralisation on access to healthcare in particular, through which we anticipate contributing to that understanding. Qualitative research is well-suited for gaining insights during implementation and understanding impacts on outcomes^10^; our qualitative study allows us to unpack the complexities of the impact of decentralisation on users’ access to healthcare from the multiple and intersecting perspectives of users, healthcare workers and administrators, to be reported in future papers.

## Ethical issues

Not applicable.

## Competing interests

Authors declare that they have no competing interests.

## Authors’ contributions

JM drafted a plan for responding to the commentaries. TA and NN contributed to the plan. JM drafted the response. JM and NN contributed to the revisions of the response.
